# Seed and Root Endophytic Fungi in a Range Expanding and a Related Plant Species

**DOI:** 10.3389/fmicb.2017.01645

**Published:** 2017-08-29

**Authors:** Stefan Geisen, Olga Kostenko, Mark C. Cnossen, Freddy C. ten Hooven, Branko Vreš, Wim H. van der Putten

**Affiliations:** ^1^Department of Terrestrial Ecology, Netherlands Institute of Ecology Wageningen, Netherlands; ^2^Laboratory of Nematology, Wageningen University Wageningen, Netherlands; ^3^Institute of Biology, Scientific Research Centre of the Slovenian Academy of Sciences and Arts Ljubljana, Slovenia

**Keywords:** endophytes, fungi, range expanding plant species, cultivation, phylogeny, soil, seeds, soil sterilization

## Abstract

Climate change is accelerating the spread of plants and their associated species to new ranges. The differences in range shift capacity of the various types of species may disrupt long-term co-evolved relationships especially those belowground, however, this may be less so for seed-borne endophytic microbes. We collected seeds and soil of the range-expanding *Centaurea stoebe* and the congeneric *Centaurea jacea* from three populations growing in Slovenia (native range of both *Centaurea* species) and the Netherlands (expanded range of *C. stoebe*, native range of *C. jacea*). We isolated and identified endophytic fungi directly from seeds, as well as from roots of the plants grown in Slovenian, Dutch or sterilized soil to compare fungal endophyte composition. Furthermore, we investigated whether *C. stoebe* hosts a reduced community composition of endophytes in the expanded range due to release from plant-species specific fungi while endophyte communities in *C. jacea* in both ranges are similar. We cultivated 46 unique and phylogenetically diverse endophytes. A majority of the seed endophytes resembled potential pathogens, while most root endophytes were not likely to be pathogenic. Only one endophyte was found in both roots and seeds, but was isolated from different plant species. Unexpectedly, seed endophyte diversity of southern *C. stoebe* populations was lower than of populations from the north, while the seed endophyte community composition of northern *C. stoebe* populations was significantly different southern *C. stoebe* as well as northern and southern *C. jacea* populations. Root endophyte diversity was considerably lower in *C. stoebe* than in *C. jacea* independent of plant and soil origin, but this difference disappeared when plants were grown in sterile soils. We conclude that the community composition of fungal endophytes not only differs between related plant species but also between populations of plants that expand their range compared to their native habitat. Our results suggest that fungal endophytes of two *Centaurea* species are not able to systemically infect plants. We highlight that endophytes remain poorly studied and further work should investigate the functional importance of endophytes.

## Introduction

Ongoing anthropogenic global climate warming has enabled many plant species to expand their natural range (Walther et al., 2002; Parmesan, 2006) leading to an increase of non-native plant species in more northern, previously unsuitable, latitudes (Tamis et al., 2005). However, plants are more or less tightly linked to numerous associated organisms, which differ in their capacity to shift their range (Berg et al., 2010). Interactions between migrating plants and associated organisms have been studied extensively on introduced exotic plant species that have moved across continents. Some of these plant species become invasive, which is often attributed to a relaxation of plant interactions with specialized natural enemies, such as fungal pathogens (Keane and Crawley, 2002; Mitchell and Power, 2003). In the past decade a number of studies have suggested that such enemy release may also occur during intracontinental range shifts (Van Grunsven et al., 2007; Engelkes et al., 2008; Morriën et al., 2010), which has been demonstrated in several cases by comparing plant responses to soil from the original and new ranges (Van Grunsven et al., 2010; Dostálek et al., 2016). However, the plant holobiome (Mitter et al., 2016) consists of a much wider range of functionally diverse organisms and the holobiome concept most adequately matches to those organisms that are in intimate symbiotic relations with plants, such as endophytes. Little is known about how plant endophyte communities may respond to climate warming-induced range shifts within continents.

Endophytes are defined as organisms that asymptotically inhabit other organisms, without causing readily visible disease symptoms as pathogens do, or promoting plant performance as arbuscular mycorrhizal fungi (AMF) do (Stone and Bacon, 2000). Among the main groups of endophytes are fungi, hereafter simplified as ‘endophytes.’ Many, if not all plant species usually host several endophyte species simultaneously (Vandenkoornhuyse et al., 2002). In contrast to AMF, the phylogenetic diversity of endophytes is, enormous and spans the entire fungal kingdom, although most endophytes belong to the phylum Ascomycota (Jumpponen and Trappe, 1998; Arnold et al., 2000; Wehner et al., 2014; Glynou et al., 2016). Many fungal endophytes are highly plant host-specific, which explains their immense diversity (U’Ren et al., 2012; Wehner et al., 2014). Consequently, endophyte communities are suggested to resemble the phylogeny of their host plant (Johnston-Monje and Raizada, 2011) more than fungi that colonize the plant surface only.

In spite of the definition suggesting that endophytes are commensals rather than enemies or symbionts, endophytes can affect plant performance, especially under stress (Newsham, 2011). For instance, endophytes increase plant performance under thermal stress (Redman et al., 2002), induce plant resistance resulting in a reduction of root feeding nematodes (Martínez-Medina et al., 2017) and herbivores (Cosme et al., 2016). Under ambient conditions, endophytes might have both positive (Newsham, 2011) or negative effects on plant performance (Mayerhofer et al., 2012; Kia et al., 2017). Effects of endophytes are likely more important under stress. In addition to their role in plant performance, many endophytes can survive and grow as saprophytes in soils (Peay et al., 2016) and include species that are primary decomposers of infected plant material (Song et al., 2017). Therefore, the exact functions of most endophyte species remains largely unknown (Newsham, 2011), whereas their role in climate warming-induced plant range shifts has been completely unstudied.

Endophyte communities differ not only between plant species, but also between plant tissues. For instance, roots host communities of endophytes that largely differ from those in stems, leafs and shoots (Fisher and Petrini, 1992; Rodriguez et al., 2009), with only few endophytes being capable of systematically infecting the host plant (Johnston-Monje and Raizada, 2011). Further, plants interact with different communities of fungi during their life time and are most strongly affected in early (seed and seedling) growth stages (Gure et al., 2005; Müller et al., 2016). Fungi reduce survival of competing plants at early plant stages, while promoting adult plants resulting in higher plant diversity (Bennett and Cahill, 2016). Most plant-endophyte studies have been performed in agricultural settings, on grasses and on trees (Schardl et al., 2004; Rodriguez et al., 2009), while the effects of fungal endophytes in wild plants have rarely been studied (Rodriguez et al., 2009; Herrera Paredes and Lebeis, 2016) as most studies have. The tight connection of endophytes and their hosts has resulted in specific coevolutionary adaptations. Many endophytes can disperse ‘vertically,’ i.e., via seeds produced by their hosts, and mutualistic seedborne endophytes can promote germination (Ernst et al., 2003; Schardl et al., 2004). However, many pathogenic organisms that use seeds as vectors for dispersal may suppress seed germination (Elmer, 2001; Schardl et al., 2004). As belowground organisms have a limited dispersal capacity (Berg et al., 2010), this might be an important strategy to spread along with their plant host, which, in case of pathogens, can reduce the success of range expanding plant species.

To study endophytes in relation to climate warming-induced range expansion of a wild plant species we isolated endophytes from the seeds of the range expanding plant species *Centaurea stoebe* and the congeneric native *Centaurea jacea*. Both plants are native in Slovenia, whereas *C. jacea* is also native in the Netherlands, which is the expanded range of *C. stoebe*. We grew the seeds in sterilized soil, as well as in sterilized soil inoculated with soils collected from Slovenia and the Netherlands, and isolated fungal endophytes from the roots of the adult plants. We tested the hypotheses that (1) seed endophytic community will resemble the core root endophytic community as seed endophytes could quickly infect germinating roots; (2) endophyte community isolated from the roots or seeds of *C. jacea* will differ from endophyte community from *C. stoebe* as plant pathogens generally are species specific; and (3) southern and northern populations of *C. jacea* will host equal number and similar communities of endophyte taxa while northern populations of the range expander *C. stoebe* will host less and distinct endophyte taxa than southern populations due to the (partial) release from plant-species specific fungi during range shift.

## Materials and Methods

### Plant Species

Climate change such as global climate warming threatens many plant species (Thomas et al., 2004) but also enables plants to expand their naturalized habitat to previously unsuitable climate zones (Hooftman et al., 2006; Van der Putten, 2012). Among them is *C. stoebe* L, a perennial in the family Asteraceae that co-occurs with the common *C. jacea* L. in riverine habitats in the Netherlands. *C. stoebe* originates from Central and South-Eastern Europe (original range) and has expanded its range to higher latitudes in the past century. It has been established in the Netherlands (new range) since 1950 (Sparrius, 2014) but it is still considered a rare species in the novel range, as it occurs only in a few locations.

### Seed Collection and Germination

In total more than 1000 seeds of more than 50 *C. jacea* and *C. stoebe* plants were collected from three randomly chosen populations in the Netherlands and Slovenia (Supplementary Table S1). From each population, 100 seeds were surface-sterilized in 5% household bleach (Dunne bleek, OKE, the Netherlands) for 3 min followed by washing with sterile demineralized H2O. Seeds were germinated on glass beads with sterile demineralized H2O in a growth cabinet at a 20/10°C; 16/8 h light/dark day/night regime under 60% humidity.

### Soil Collection

Soil was collected from three different locations within a riverine area in Slovenia (35 kg; N45° 58′- 46° 09′; E014° 32′- E014° 45′) and the Netherlands (500 kg; N51° 51′; E5° 53′; Supplementary Table S2). The higher amount of soil needed from the Netherlands was because we only inoculated 10% alive soil (either from Slovenia or the Netherlands to 90% sterile soils (see below for further details). The soil was collected from 5 to 20 cm layer below the soil surface and transported cool (4°C) to the laboratory. In the laboratory, soil was sieved through a 5 mm × 5 mm mesh to remove larger stones, insect larvae, and earthworms. Then, the individual soil samples were formed by pooling three soil subsamples from the same riverine area. All pooled soils were sterilized by autoclaving (high pressure saturated steam at 121°C for 20–40 min) three times with 24 h interval. The live soils were kept in the dark climate room (4°C) before the use in the experiment.

### Greenhouse Experiment

To create soil treatments 36 1-L pots were filled with a mix of live and sterilized Dutch soil (1:9; NL soil treatment); 36 pots were filled with a mix of live Slovenian and sterilized Dutch soil (1:9; SLO soil treatment); 36 pots were filled with a mix of sterilized Slovenian and Dutch soils (1:19; STERILE soil treatment). All pots received the same amount of soil (950 g) calculated based on dry weight of different soils. Germinated plant seedlings of similar size were individually planted in each pot. The pots were randomly placed on movable carts in a greenhouse at 21/15°C; 16/8 h light/dark; 60% humidity. Soil moisture was kept constant throughout the experiment by adjusting the pot weight to 1 kg with sterile demineralized H_2_O every second day. The carts were rotated weekly to avoid effects of variable conditions in the greenhouse. Rarely germinating weeds (in the live soil treatments) were instantly removed. No nutrients were added to the pots.

Eleven weeks after planting seedlings, shoots of all plants were clipped. Roots were carefully removed from the soil and thoroughly rinsed. Then, five randomly collected root fragments of approximately 5 cm length each were cut into pieces of 0.5 cm and stored in 2 mL centrifuge tubes filled with sterilized demineralized H_2_O.

### Isolation of Fungal Endophytes from Seeds

To cultivate fungi, three seeds of all populations from both plant species (Supplementary Table S1) were placed on 1.6% H_2_O-agar pH 6.7 containing 50 μg/ml streptomycin in 10 cm Petri dishes. The seeds were first surface sterilized using two sterilization protocols; (1) rinsing with sterile demineralized H_2_O for 5 min; (2) thoroughly sterilizing by soaking seeds in 5% household bleach solution for 5 min followed by incubation in 70% ethanol for 3 min and washing with sterile demineralized H_2_O. For each plant species, five replicates per population with were initiated. The resulting cultures of endophytes were transferred to 0.5x potato-dextrose agar pH 6.7 (PDA; Oxoid) (Larone, 1987).

We also used another procedure aiming at isolating oomycetes. For that, five rinsed and thoroughly surface sterilized seeds (as explained before) of all populations from both plant species (Supplementary Table S1) were placed in 6 cm Petri dishes filled with a mix of sterile pond water filtered through cheesecloth and sterile demineralized H_2_O (1:1) containing grass leaves (*Agrostis capillaris*, 2–3 cm) to bait zoospore forming oomycetes (Pettitt et al., 2002). After incubation overnight at room temperature, grass leafs were transferred on 1.6% H_2_O-agar pH 6.7 containing 50 μg/ml streptomycin in a 6 cm diameter Petri dish. Growing cultures were transferred to a 6 cm diameter Petri dish containing 0.5x potato-dextrose agar pH 6.7 (PDA; Oxoid). To reduce the number of potentially duplicated isolates for both fungi and oomycetes, only one culture isolated from the same plant replicate was kept in case they were morphologically indistinguishable (Bosshard, 2011).

### Isolation of Fungal Endophytes from Roots

To surface sterilize the roots, the collected root pieces were thoroughly washed in sterile demineralized H_2_O, transferred to new sterilized centrifuge tubes filled with 70% ethanol and incubated for 7 min. Root pieces were washed again in sterile demineralized H_2_O, and then placed in sterile demineralized H_2_O in new centrifuge tubes, followed by surface drying on sterile tissue paper under sterile conditions in a flow cabinet. Three root pieces were placed on 1.6% H_2_O-agar pH 6.7 containing 50 μg/ml streptomycin in 10 cm diameter Petri dishes. Five replicates were initiated and stored at room temperature.

The remaining root pieces were divided into three and placed into a 6 cm diameter Petri dishes filled with a mix of sterile pond water filtered through cheesecloth and sterile demineralized H_2_O (1:1) together with three grass leafs (*Agrostis capillaris*, 2–3 cm). The Petri dishes were placed at room temperature, incubated overnight. Grass leaves were transferred onto a 10 cm Petri dish containing 1.6% H_2_O-agar pH 6.7 containing 50 μg/ml streptomycin. All H_2_O-agar containing Petri dishes were checked for fungal and oomycete growth and newly formed colonies were transferred to Petri dishes containing 0.5x potato-dextrose agar pH 6.7 (PDA; Oxoid) (Larone, 1987). To reduce the number of potentially duplicated isolates, only one culture isolated from the same plant replicate was kept in case they were morphologically indistinguishable (Bosshard, 2011).

### Molecular Work

DNA was extracted from all cultures using the Zymo Research Fungal/Bacterial miniprep kit according to the manufacturer’s instructions. The ITS region of all cultures was PCR amplified in 25 μl volume containing 3.125 μL 2 mM dNTPs, 1 μL 25 mM MgCl_2_, 2.5 μL 10x buffer with MgCl_2_, 0.125 μL 5 U [μL]-1 FastStart Taq DNA polymerase (Roche Diagnostics GmbH, Mannheim, Germany), 15.25 μL ddH_2_O, 1 μL 10 μM of both primers ITS1 and ITS4 (White et al., 1990) and 1 μL template DNA. PCR conditions were composed of an initial denaturation at 95°C for 10 min, followed by 40 cycles of denaturation at 95°C for 30 s, annealing at 56°C for 30 s and elongation at 72°C for 60 s with a final elongation at 72°C for 10 min. Amplified products were send for sequencing (LGC Genomics, Berlin, Germany).

### Sequence Analyses

Obtained chromatograms from all cultures were manually curated in Chromas Lite v 2.1^1^. Curated sequences were aligned using MAFFT (Katoh and Standley, 2013) and visualized in Seaview v4.6.1 (Gouy et al., 2010).

The resulting 46 consensus sequences were subjected to BLASTn searched against the NCBI nucleotide database^2^. For all consensus sequences, two best matches of known fungal species (three in case different species showed identical matches to best Blast matches of cultivated taxa, or unknown sequences), were aligned using MAFFT and visualized in Seaview. This resulted in an alignment containing 130 sequences.

Maximum likelihood (ML) and Bayesian analyses were performed to assess the phylogenetic relatedness of all cultivated fungi. Maximum likelihood analyses were run in RAxML v8. (Stamatakis, 2014) using the GTR+gamma model with eight rate categories. Rapid hill-climbing tree search algorithm with 1000 bootstrap replicates were used to build and assess the most stable shape of phylogenetic relationships. The phylogenetic tree was visualized in FigTree (Rambaut, 2007) and labeling of the final branches optimized in Gimp.

### Data Depository

All sequence data has been submitted to GenBank under the accession numbers MF687671–MF687716.

### Data Analyses

For seed endophyte data the total number of unique cultures was calculated per Petri dish (*n* = 24, one Petri dish per one treatment combination) and this data were used for the analyses. For root endophyte data the total number of unique cultures (cultures with distinct sequences) was calculated per pot (*n* = 108, three pots per one treatment combination). Therefore, to avoid pseudoreplication we first averaged the data per treatment combination and used this data for further analyses. To test the effect of plant species and seed origin on the number of seed endophyte cultures that were identified as being taxonomically different, we used general linear model with plant species (*C. jacea* and *C. stoebe*), seed origin (*North* – collected in The Netherlands and *South* – collected in Slovenia) and sterilization treatment (non-sterilized and sterilized) as fixed factors. Differences in the number of root cultures between plant species, seed and soil origins were analyzed using general linear model with plant species (*C. jacea* and *C. stoebe*), seed origin (*North* and *South*) and type of soil inoculum (Dutch soil -*NL*, Slovenian soil -*SLO* and *Sterile soil*) as fixed factors. In both analyses, populations were treated as true replicates.

To test whether the seed and root endophyte community composition was affected by plant species, seed origin and type of soil inoculum we used detrended correspondence analyses (DCA) and canonical correspondence analyses (CCA). For this we used presence-absence data of endophyte cultures. Detrending by segments was used in the DCA. Populations were treated as true replicates. For seed endophyte community analyses, all endophytes were included in the analyses. For root endophyte community analyses, the endophytes with more than two occurrences were included in the analyses because there were a large number of endophytes with one occurrence only.

Multivariate analyses were performed using CANOCO, version 5.03 (Šmilauer and Lepš, 2014) and all other analyses were executed using R, version 3.2.3 (R Core Team, 2015). To fulfill the assumptions of normality and homogeneity of variances total number of root endophyte cultures was log-transformed.

## Results

### Taxonomic Diversity of Endophytes

After removing morphologically and phylogenetically identical species from the respective plant populations grown in southern and northern soils, we obtained 91 distinct seed and 72 unique root endophytes. Many of those shared identical sequences resulting in 46 unique sequences (Supplementary Table S3).

**FIGURE 1:**
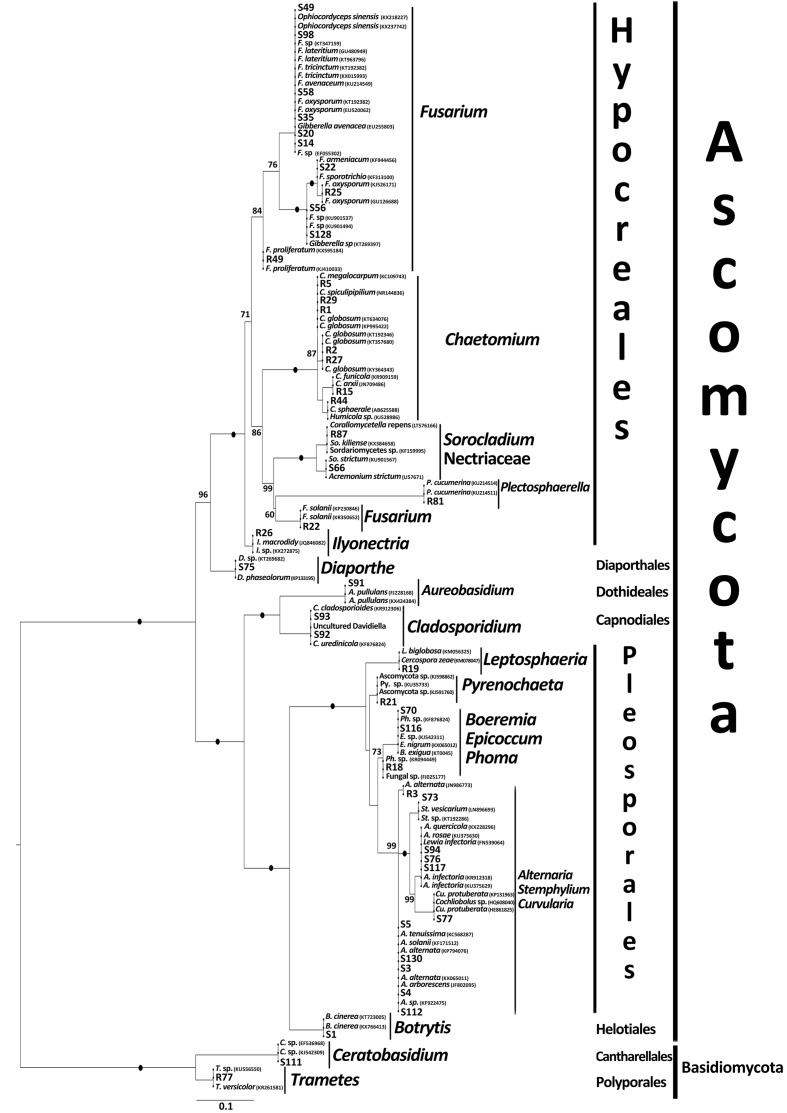
Maximum likelihood phylogenetic tree of all cultivated fungal endophytes (bold) and their best blast matches showing their phylogenetic affinities. For additional details see Supplementary Table S3.

All cultures except two basidiomycetes resembled fungi of the Ascomycota (**Figure 1** and Supplementary Table S3). The 46 unique sequences were phylogenetically diverse and placed in 19 genera, 15 families, 10 orders, and 4 fungal classes (**Figure 1** and Supplementary Table S3).

### Comparison between the Species Identities and Community Composition of Seed and Root Endophytes

The surface sterilization we used was highly efficient as on average less than one different culture (0.67) was obtained from inside roots, while sterilized seeds harbored more cultures (3.79) per population. The seed endophyte community yielded 28 unique cultures, whereas the root endophyte community yielded 17 unique cultures. There was no overlap in endophyte composition of roots and seeds, besides one culture with perfect match to *Fusarium oxysporum* f. sp. *cumini* that was collected from southern *C. stoebe* seeds, as well as from southern *C. jacea* roots (Supplementary Table S3).

### Plant Species, Seed Origin, and Sterilization Effects on the Diversity and Community Composition of Seed Endophytes

Potential pathogens dominated the community composition of seed endophytes, with especially *Fusarium* and *Alternaria* species representing more than 50% of the different isolates (Supplementary Table S3).

**FIGURE 2:**
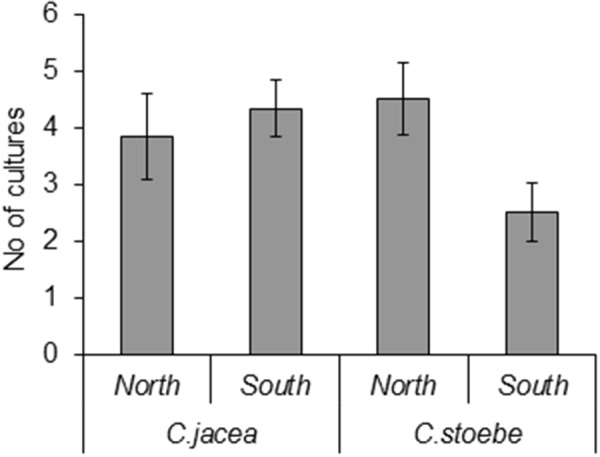
Numbers of different fungal endophyte taxa isolated from southern and northern seeds of *Centaurea jacea*, which is native in both ranges, and the range expander *Centaurea stoebe*, which is native in the south.

Seed endophyte taxa richness was significantly affected by the interaction between plant species and seed origin (*F*_1,16_ = 5.23, *P* = 0.036; **Figure 2**). In particular, the number of cultures isolated from the *C. stoebe* seeds collected in the southern range was lower than in the northern range whereas the number of cultures isolated from the *C. jacea* seeds did not differ between origins (**Figure 2**).

**FIGURE 3:**
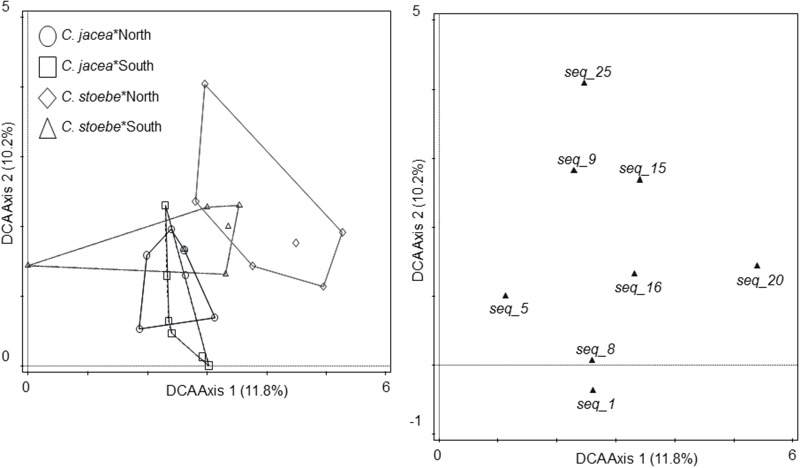
Ordination diagrams of detrended correspondence analysis (DCA) of endophyte community isolated from seeds of *C. jacea* collected in The Netherlands (“North,” circles) and in Slovenia (“South,” squares), as well as seeds of *C. stoebe* collected in The Netherlands (“North,” diamonds) and in Slovenia (“South,” open triangles, left panel). All endophyte cultures with more than 10% fit are shown (right panel). Percentages of total explained variation by DCA axes are given in parentheses.

The community composition of seed endophytes was affected by the two-way interactions between plant species and seed origin (*F* = 1.5; *P* = 0.001; 18.3% explained variation, adjusted explained variation 6.0%; **Figure 3**). Seed sterilization did not affect the community composition of endophytes (*F* = 0.6; *P* = 0.997).

### Plant Species, Seed Origin, and Soil Origin Effects on the Diversity and Community Composition of Root Endophytes

**FIGURE 4:**
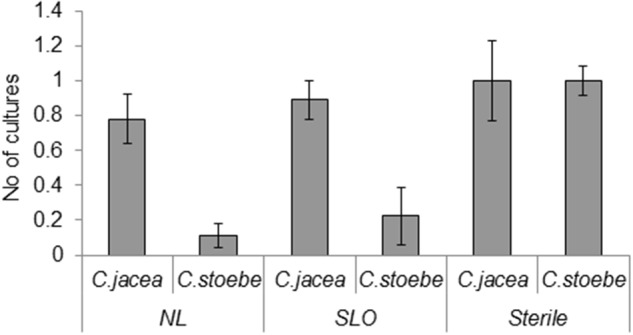
Numbers of different fungal endophyte taxa isolated from roots from the common *C. jacea* and the range expander *C. stoebe* grown in northern (NL), southern (SLO), and sterile soils (Sterile) expander *C. stoebe*.

**FIGURE 5:**
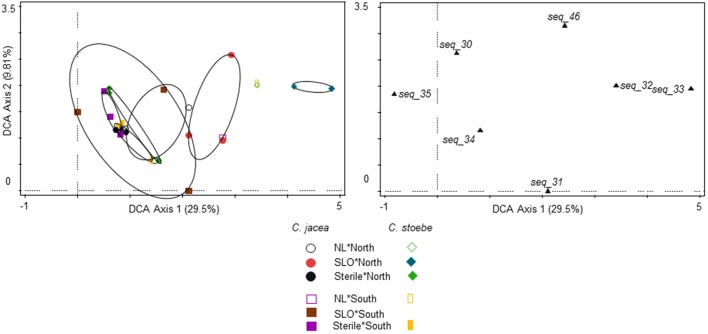
Ordination diagrams of DCA of endophyte community isolated from roots of northern *C. jacea* plants grown in northern (open black circles), southern (filled red circles), and sterilized soils (filled black circles); from roots of southern *C. jacea* plants grown in northern (open pink squares), southern (brown filled squares), and sterile soils (pink filled squares); from roots of northern *C. stoebe* plants grown in northern (open green diamonds), southern (filled blue diamonds), and sterile soils (filled green diamonds); from roots of southern *C. stoebe* plants grown in northern (open yellow rectangles) and sterile soils (filled yellow rectangles). All endophyte cultures with are shown (right panel). Percentages of total explained variation by DCA axes are given in parentheses. The overlapping symbols have been shifted by 0.1 unit to improve the visibility.

The most common (present in more than 10% of the samples) root endophytes were *Chaetomium* spp. (Supplementary Table S3). Range-expanding *C. stoebe* hosted a lower diversity of root endophytes than *C. jacea* plants in NL and Slo soils whereas in sterilized soil there was no difference in the diversity of root endophytes between the two plants species (*F*_2,24_ = 7.71, *P* = 0.0026; **Figure 4**). Root endophyte community composition was affected by a combination of plant species, soil and seed origin when singleton endophytes were not included in the analyses (by three-way interaction; *F* = 2.4; *P* = 0.005, 61.1% explained variation; 35.2% adjusted explained variation; **Figure 5**).

## Discussion

We here show that there is no overlap of root-inhabiting and seed-inhabiting fungal endophytes in neither the range expanding plant species *C. stoebe* nor its common congener *C. jacea*. This strongly suggests that seeds might not serve as vehicles for (pathogenic) root endophytes to spread to new ranges.

### High Diversity of Mainly Ascomycete Fungal Endophytes in Seeds and Roots

Almost all endophytes cultivated from both seeds and roots belong to Ascomycota, which is in line with many previous studies on endophytes in different parts of the plant (Jumpponen and Trappe, 1998; Arnold et al., 2000; Rodriguez et al., 2009). Interestingly, most seed endophytes, in contrast to root endophytes, most closely resembled potential pathogens. The overall function of endophytes is being debated (Newsham, 2011; Mayerhofer et al., 2012) and remains largely unknown (Vandenkoornhuyse et al., 2002). We assigned potential functions based on sequence identity without experimentally testing the vast amount of cultures. This approach is well-accepted to get an overall understanding of likely functions (Nguyen et al., 2016). Therefore, our results suggest that endophytes differ in their functioning between infected plant tissues with most endophytes inhabiting seeds likely being negative while root endophytes being neutral or positive for plant performance.

### No Overlap between Seed and Root Endophytes

In line with potential functional differences between seed and root endophytes, seed and roots hosted a fundamentally different community of endophytes. Only one sequence resembling *F. oxysporum* f. sp. *cumini* was shared between an endophyte isolated from a seed and another one isolated from the root of southern plants. The presence of this fungi is not surprising as *F. oxysporum* f. sp. *cumini* generally has the ability to infect and damage host roots and shoots (Özer and Bayraktar, 2015). However, differentiating *F. oxysporum* subspecies and especially races is difficult and relies on other markers than the one we used here (ITS) as a general fungal barcode (Gordon and Martyn, 1997; Lievens et al., 2008). Considering that even races within *F. oxysporum* f. sp. *cumini* are differentially impacting plant performance (Özer and Bayraktar, 2015) and the fact that the sequence-identical cultures we obtained were originating from cultures isolated from two different plant species, ecological function of both cultures could be dissimilar. This further shows that root and seed endophyte communities are fundamentally different, suggesting that most endophytes have a restricted localization in the plant tissues in at least in the *Centaurea* species studied here (Rodriguez et al., 2009). The restriction of belowground endophytic fungi to root tissue also reduces the possibility that these belowground taxa spread (Berg et al., 2010). This would give the plant a competitive advantage in case they escape their more specialized pathogens – some of which are considered as being endophytic (Saikkonen et al., 1998).

### Seed Endophytes Differ in *C. stoebe* Plants in the Expanded Range

Species specific seed endophytes seem to be generally plant pathogenic, which could control plant growth and in turn population dynamics, independent whether the plant is native or not (Blaney and Kotanen, 2001). Interestingly, we found the lowest diversity of (potentially pathogenic) fungi in the native range of *C. stoebe* compared to all other plant seed populations including its expanded range, while endophyte diversity in seeds of *C. stoebe* in the expanded range was highest. Fungi that are more generalist and less pathogenic might therefore infect seeds in the expanded range that have less negative impact on plant growth, which is in line with fungi having more negative effect on plant seeds in the native compared to the invaded range (Halbritter et al., 2012). These pathogenic endophytes could therefore be among the “enemies” that drive range expansion according to the enemy release hypothesis, which is among the key hypotheses to explain the success of plants coming to a new range (Keane and Crawley, 2002; Reinhart et al., 2003). These results indicate that *C. stoebe* cannot only expand, but can only perform well in new ranges indirectly by “escaping” their associated endophytes. While endophytes are directly impacted by climate change (Giauque and Hawkes, 2013) and soil biota are known to affect the performance of range expanding plants (Van Nuland et al., 2017), we lack an integrated understanding on the importance of endophytes in climate induced range expansions. Functional studies on a range of endophytes and their hosts in both ranges are needed to confirm that this hypothesis equally holds for seed endophytes.

### Root Endophytes

In contrast to seed endophytes, root endophytes were mostly assigned as being non-pathogenic. Still, and in line with the seed endophytes, the diversity of root endophytes was much lower in *C. stoebe* than in *C. jacea* when plants were grown in Dutch or Slovenian soils. This suggests that compared with *C. jacea*, the range expanding plant *C. stoebe* is more antagonistic toward soil organisms as previously shown for fungi and root feeding nematodes (Wilschut et al., 2016), but nematodes might not always be reduced (Morriën et al., 2012; Viketoft and van der Putten, 2015). *C. stoebe* is known to produce a wide range of secondary metabolites that are more negative toward soil organisms in a new range, making it a noxious invader, especially in the United States, where it threatens natural systems (Ortega and Pearson, 2005; Ridenour et al., 2008). Production of secondary metabolites might be the key underlying factor that acts against plant-specialized soil pathogenic organisms and act as a novel weapon to change the microbial community structure to their own favor (Callaway et al., 2008; Verhoeven et al., 2009). This would also lead to a reduced infection potential of fungal endophytes as observed here.

In line with the reduction of seed endophytes in southern seeds of *C. stoebe*, no endophytes were cultured when those seeds where grown in southern soils. This suggests that southern seeds of *C. stoebe* seem particularly strong in defending themselves especially in their native soil habitat. This allows *C. stoebe* to defend against specialized native pathogens and, when expanding, benefit from more general, less harmful interactions, which is supported by a profound increase in infection in sterilized soils. Northern plants which escaped specialized pathogen pressure decades ago host higher endophyte diversities suggesting a trade-off toward losing costly-to-produce chemical defenses in favor of growth, which also allows them to benefit from more generalist mutualistic interactions including AMF (Bunn et al., 2015) and potentially endophytes as suggested here. Furthermore, range expanding plants could benefit in plant communities by accumulating pathogens that are deleterious for native plants (Mangla and Callaway, 2008). This suggests that range expanding plants benefit initially mostly from a release of specialist pathogens, while later from other mechanisms including increase of mutualists.

The profound reduction of endophytes in *C. stoebe* compared with *C. jacea* that is lost in sterile soil suggests that the native rhizosphere microbiome represents an intimate component of the plants ability to cope with incoming organisms. This is in line with more reduced mechanistic studies that show that invasive microbes are less likely to establish when a more diverse naïve microbiome is present (Mallon et al., 2015; Wei et al., 2015). This result further suggests that experiments done in sterile soils under non-sterile conditions can miss patterns that are actually present under more natural conditions. We therefore propose to avoid using experimental setups with entirely sterilized soils and always inoculate at least a fraction of natural soil to reduce the random effects of invasive microorganisms.

## Conclusion

We conclude that there is no overlap in the taxonomic composition of endophytes in seeds and roots in the range expanding (*C. stoebe*) and its common congener (*C. jacea*), suggesting that root-inhabiting organisms, including plant pathogens, cannot spread along with the plant to infect roots in the new range. We further show that the range expanding plant hosts a reduced diversity of endophytes in roots, but that this difference is not present when soils are sterilized. This suggests that range expanding plants only in combination with a diverse microbiome obtained from soils have an increased defense against specialized soilborne organisms including pathogens, which provides them with a competitive advantage to establish in plant communities. Overall the distribution of endophytes in different plant species, especially their presence in different plant parts remains little studied. Moreover, there are no studies focusing on endophytes in range expanding plant species. As these can adapt distinct ecological roles their functional importance to affect range expanding plant species remains to be elucidated.

## Author Contributions

SG, OK, and WP designed the study; BV, MC, FH, and SG conducted the experimental work, SG and OK analyzed data; SG, MC, OK, and WP drafted the manuscript supplemented with comments from FH.

## Conflict of Interest Statement

The authors declare that the research was conducted in the absence of any commercial or financial relationships that could be construed as a potential conflict of interest.

## Footnotes

1 http://chromas-lite.software.informer.com/2.1/

2 http://www.ncbi.nlm.nih.gov/Blast.cgi
