# Supplementary material for: Seed and Root Endophytic Fungi in a Range Expanding and a Related Plant Species
Source: Front Microbiol. 2017 Aug 29;8:1645. doi: 10.3389/fmicb.2017.01645 (PMC5581836; doi:10.3389/fmicb.2017.01645)
Supplement: Supplementary file 1 [file Table_1.DOCX]

Table S1. Sites were the seeds of *Centaurea jacea* and *C. stoebe* were collected.

| Plant species | Seed origin | Population | Sampling site | Coordinates (North; East) |
| --- | --- | --- | --- | --- |
| *C. jacea* | north | 1 | The Netherlands | 51°52'45.8"; 6°00'16.9" |
| *C. jacea* | north | 2 | The Netherlands | 51°51'55.68"; 6°0'44.37" |
| *C. jacea* | north | 3 | The Netherlands | 51°52'04.9"; 5°59'32.5" |
| *C. jacea* | south | 4 | Slovenia | 46°08'13.54"; 014°36'58.32" |
| *C. jacea* | south | 5 | Slovenia | 46°09'91.62"; 014°45'33.90" |
| *C. jacea* | south | 6 | Slovenia | 46°22'37.64"; 014°10'06.62" |
| *C. stoebe* | north | 7 | The Netherlands | 50°58'19.2"; 5°59'16.8" |
| *C. stoebe* | north | 8 | The Netherlands | 51°49'54.7"; 4°7'54.5" |
| *C. stoebe* | north | 9 | The Netherlands | 51°51'60.3"; 5°53'33.6" |
| *C. stoebe* | south | 10 | Slovenia | 45°55'58.10"; 015°29'78.10" |
| *C. stoebe* | south | 11 | Slovenia | 45°55'73.46"; 015°30'50.88" |
| *C. stoebe* | south | 12 | Slovenia | 45°55'82.28"; 015°29'74.02" |
