# Supplementary material for: Seed and Root Endophytic Fungi in a Range Expanding and a Related Plant Species
Source: Front Microbiol. 2017 Aug 29;8:1645. doi: 10.3389/fmicb.2017.01645 (PMC5581836; doi:10.3389/fmicb.2017.01645)
Supplement: Supplementary file 2 [file Table_2.DOCX]

Table S2. Soil collection sites

| Soil origin | Sampling area | Sub-sample within sampling area | Coordinates (North; East) |
| --- | --- | --- | --- |
| north | The Netherlands. Vicinity of the river Waal | 1 | N51° 51.511' E5° 53.146' |
| north |  | 2 | N51° 51.538' E5° 53.133' |
| north |  | 3 | N51° 51.536' E5° 53.092' |
| south | Slovenia, vicinity of the river Sava | 1 | N46° 08' 08.124'' E014° 36' 34.992'' |
| south |  | 2 | N46° 09' 54.972'' E014° 45' 20.340'' |
| south |  | 3 | N45° 58' 08.544'' E014° 32' 44.592'' |
