# Supplementary material for: Seed and Root Endophytic Fungi in a Range Expanding and a Related Plant Species
Source: Front Microbiol. 2017 Aug 29;8:1645. doi: 10.3389/fmicb.2017.01645 (PMC5581836; doi:10.3389/fmicb.2017.01645)
Supplement: Supplementary file 3 [file Table_3.DOCX]

Table S3. Unique endophyte cultures enriched in the experiment and their likely function based on best Blast hits and literature information.

| **Name** | **Division** | **Class** | **Order** | **Familiy** | **Genus** | **Species** | **Likely function** |
| --- | --- | --- | --- | --- | --- | --- | --- |
| S22 | Ascomycota | [Sordariomycetes](https://en.wikipedia.org/wiki/Sordariomycetes) | [Hypocreales](https://en.wikipedia.org/wiki/Hypocreales) | [Nectriaceae](https://en.wikipedia.org/wiki/Nectriaceae) | [*Fusarium*](https://blast.ncbi.nlm.nih.gov/Blast.cgi#alnHdr_576941508) | *F. armeniacum/sporotrichioides* | plant pathogen |
| S128 | Ascomycota | [Sordariomycetes](https://en.wikipedia.org/wiki/Sordariomycetes) | [Hypocreales](https://en.wikipedia.org/wiki/Hypocreales) | [Nectriaceae](https://en.wikipedia.org/wiki/Nectriaceae) | [*Fusarium*](https://blast.ncbi.nlm.nih.gov/Blast.cgi#alnHdr_576941508) | *F. graminearum* | plant pathogen |
| S56 | Ascomycota | [Sordariomycetes](https://en.wikipedia.org/wiki/Sordariomycetes) | [Hypocreales](https://en.wikipedia.org/wiki/Hypocreales) | [Nectriaceae](https://en.wikipedia.org/wiki/Nectriaceae) | [*Fusarium*](https://blast.ncbi.nlm.nih.gov/Blast.cgi#alnHdr_576941508) | *F. sp (graminearum-like)* | plant pathogen |
| S20 | Ascomycota | [Sordariomycetes](https://en.wikipedia.org/wiki/Sordariomycetes) | [Hypocreales](https://en.wikipedia.org/wiki/Hypocreales) | [Nectriaceae](https://en.wikipedia.org/wiki/Nectriaceae) | [*Fusarium*](https://blast.ncbi.nlm.nih.gov/Blast.cgi#alnHdr_1002614778) | *F. lateritium* | plant pathogen |
| S14 | Ascomycota | [Sordariomycetes](https://en.wikipedia.org/wiki/Sordariomycetes) | [Hypocreales](https://en.wikipedia.org/wiki/Hypocreales) | [Nectriaceae](https://en.wikipedia.org/wiki/Nectriaceae) | [*Fusarium*](https://blast.ncbi.nlm.nih.gov/Blast.cgi#alnHdr_1002614778) | *F. tricinctum/acuminatum/lateritium* | plant pathogen |
| S35 | Ascomycota | [Sordariomycetes](https://en.wikipedia.org/wiki/Sordariomycetes) | [Hypocreales](https://en.wikipedia.org/wiki/Hypocreales) | [Nectriaceae](https://en.wikipedia.org/wiki/Nectriaceae) | [*Fusarium*](https://blast.ncbi.nlm.nih.gov/Blast.cgi#alnHdr_1002614778) | *F. tricinctum/acuminatum/lateritium* | plant pathogen |
| S58 | Ascomycota | [Sordariomycetes](https://en.wikipedia.org/wiki/Sordariomycetes) | [Hypocreales](https://en.wikipedia.org/wiki/Hypocreales) | [Nectriaceae](https://en.wikipedia.org/wiki/Nectriaceae) | [*Fusarium*](https://blast.ncbi.nlm.nih.gov/Blast.cgi#alnHdr_1002614778) | *F. oxysporum* | plant pathogen |
| S49 | Ascomycota | [Sordariomycetes](https://en.wikipedia.org/wiki/Sordariomycetes) | [Hypocreales](https://en.wikipedia.org/wiki/Hypocreales) | [Nectriaceae](https://en.wikipedia.org/wiki/Nectriaceae) | [*Fusarium*](https://blast.ncbi.nlm.nih.gov/Blast.cgi#alnHdr_1002614778) | *F. oxysporum* | plant pathogen |
| S98 | Ascomycota | [Sordariomycetes](https://en.wikipedia.org/wiki/Sordariomycetes) | [Hypocreales](https://en.wikipedia.org/wiki/Hypocreales) | [Nectriaceae](https://en.wikipedia.org/wiki/Nectriaceae) | [*Fusarium*](https://blast.ncbi.nlm.nih.gov/Blast.cgi#alnHdr_1002614778) | *F. tricinctum/acuminatum* | plant pathogen |
| S75 | Ascomycota | [Sordariomycetes](https://de.wikipedia.org/wiki/Sordariomycetes) | [Diaporthales](https://de.wikipedia.org/wiki/Diaporthales) | Diaporthaceae | [*Diaporthe*](https://blast.ncbi.nlm.nih.gov/Blast.cgi#alnHdr_807059227) | *D. phaseolorum* | plant pathogen |
| S1 | Ascomycota | [Leotiomycetes](https://en.wikipedia.org/wiki/Leotiomycetes) | [Helotiales](https://en.wikipedia.org/wiki/Helotiales) | [Sclerotiniaceae](https://en.wikipedia.org/wiki/Sclerotiniaceae) | [*Botrytis*](https://en.wikipedia.org/wiki/Botrytis_(fungus)) | *B. cinerea* | plant pathogen |
| S92 | Ascomycota | [Dothideomycetes](https://en.wikipedia.org/wiki/Dothideomycetes) | [Capnodiales](https://en.wikipedia.org/wiki/Capnodiales) | [Davidiellaceae](https://en.wikipedia.org/wiki/Davidiellaceae) | *Cladosporium* | *Cladosporium spp* | potential plant pathogen |
| S93 | Ascomycota | [Dothideomycetes](https://en.wikipedia.org/wiki/Dothideomycetes) | [Capnodiales](https://en.wikipedia.org/wiki/Capnodiales) | [Davidiellaceae](https://en.wikipedia.org/wiki/Davidiellaceae) | *Cladosporium* | *Cladosporium spp* | potential plant pathogen |
| S91 | Ascomycota | Dothideomycetes | [Dothideales](https://en.wikipedia.org/wiki/Dothideales) | [Aureobasidiaceae](https://en.wikipedia.org/w/index.php?title=Aureobasidiaceae&action=edit&redlink=1) | [*Aureobasidium*](https://en.wikipedia.org/w/index.php?title=Aureobasidium&action=edit&redlink=1) | *A. pullulans* | plant associated (endophytic or pathogen) |
| S4 | Ascomycota | Dothideomycetes | Pleosporales | Pleosporaceae | *Alternaria* | *A. spp* | potential plant pathogen |
| S2 | Ascomycota | Dothideomycetes | Pleosporales | Pleosporaceae | *Alternaria* | *A. alternata/solani* | potential plant pathogen |
| S3 | Ascomycota | Dothideomycetes | Pleosporales | Pleosporaceae | *Alternaria* | *A. alternata/solani* | potential plant pathogen |
| S86 | Ascomycota | Dothideomycetes | Pleosporales | Pleosporaceae | *Alternaria* | *A. alternata/solani* | potential plant pathogen |
| S112 | Ascomycota | Dothideomycetes | Pleosporales | Pleosporaceae | *Alternaria* | *A. alternata/solani* | potential plant pathogen |
| S48 | Ascomycota | Dothideomycetes | Pleosporales | Pleosporaceae | *Alternaria* | *A. infectoria/quercicola* | potential plant pathogen |
| S117 | Ascomycota | Dothideomycetes | Pleosporales | Pleosporaceae | *Alternaria* | *A. infectoria* | potential plant pathogen |
| S76 | Ascomycota | Dothideomycetes | Pleosporales | Pleosporaceae | *Alternaria* | *A. rosea* | plant endophyte |
| S73 | Ascomycota | Dothideomycetes | Pleosporales | Pleosporaceae | *Stemphylium* | *S. vesicarium* | plant pathogen |
| S116 | Ascomycota | Dothideomycetes | Pleosporales | Didymellaceae | *Boeremia* | *B. exigua* | plant pathogen |
| S70 | Ascomycota | Dothideomycetes | Pleosporales | Didymellaceae | *Epicoccum* | *E. nigrum* | plant mutualist |
| S111 | Basidiomycota | Agaricomycetes | Cantharellales | Ceratobasidiaceae | *Ceratobasidium* | *Ceratobasidium sp* | potential plant pathogen |
| S66 | Ascomycota | [Sordariomycetes](https://ceb.wikipedia.org/wiki/Sordariomycetes) | [Hypocreales](http://www.mycobank.org/BioloMICS.aspx?TableKey=14682616000000067&Rec=92466&Fields=All) | incertae sedis | *Sarocladium* | *S. strictum* | plant associated (endophytic or pathogen) |
| s77 | Ascomycota | Dothideomycetes | Pleosporales | Pleosporaceae | *Curvularia* | *C. protuberata* | plant mutualist |
| **R25_S97** | Ascomycota | [Sordariomycetes](https://en.wikipedia.org/wiki/Sordariomycetes) | [Hypocreales](https://en.wikipedia.org/wiki/Hypocreales) | [Nectriaceae](https://en.wikipedia.org/wiki/Nectriaceae) | [*Fusarium*](https://blast.ncbi.nlm.nih.gov/Blast.cgi#alnHdr_1002614778) | *F. oxysporum* | plant pathogen |
| R44 | Ascomycota | [Sordariomycetes](https://sv.wikipedia.org/wiki/Sordariomycetes) | [Sordariales](https://sv.wikipedia.org/wiki/Sordariales) | [Chaetomiaceae](https://sv.wikipedia.org/wiki/Chaetomiaceae) | *Chaetomium* | *C. sphaerale* | plant endophyte |
| R5 | Ascomycota | [Sordariomycetes](https://sv.wikipedia.org/wiki/Sordariomycetes) | [Sordariales](https://sv.wikipedia.org/wiki/Sordariales) | [Chaetomiaceae](https://sv.wikipedia.org/wiki/Chaetomiaceae) | *Chaetomium* | *C. spp* | plant endophyte |
| R1 | Ascomycota | [Sordariomycetes](https://sv.wikipedia.org/wiki/Sordariomycetes) | [Sordariales](https://sv.wikipedia.org/wiki/Sordariales) | [Chaetomiaceae](https://sv.wikipedia.org/wiki/Chaetomiaceae) | *Chaetomium* | *C. spp2* | plant endophyte |
| R29 | Ascomycota | [Sordariomycetes](https://sv.wikipedia.org/wiki/Sordariomycetes) | [Sordariales](https://sv.wikipedia.org/wiki/Sordariales) | [Chaetomiaceae](https://sv.wikipedia.org/wiki/Chaetomiaceae) | *Chaetomium* | *C. globosum* | plant endophyte |
| R2 | Ascomycota | [Sordariomycetes](https://sv.wikipedia.org/wiki/Sordariomycetes) | [Sordariales](https://sv.wikipedia.org/wiki/Sordariales) | [Chaetomiaceae](https://sv.wikipedia.org/wiki/Chaetomiaceae) | *Chaetomium* | *C. globosum* | plant endophyte |
| R27 | Ascomycota | [Sordariomycetes](https://sv.wikipedia.org/wiki/Sordariomycetes) | [Sordariales](https://sv.wikipedia.org/wiki/Sordariales) | [Chaetomiaceae](https://sv.wikipedia.org/wiki/Chaetomiaceae) | *Chaetomium* | *C. globosum* | plant endophyte |
| R49 | Ascomycota | [Sordariomycetes](https://en.wikipedia.org/wiki/Sordariomycetes) | [Hypocreales](https://en.wikipedia.org/wiki/Hypocreales) | [Nectriaceae](https://en.wikipedia.org/wiki/Nectriaceae) | [*Fusarium*](https://blast.ncbi.nlm.nih.gov/Blast.cgi#alnHdr_1002614778) | *F. oxysporum* | plant pathogen |
| R22 | Ascomycota | [Sordariomycetes](https://en.wikipedia.org/wiki/Sordariomycetes) | [Hypocreales](https://en.wikipedia.org/wiki/Hypocreales) | [Nectriaceae](https://en.wikipedia.org/wiki/Nectriaceae) | [*Fusarium*](https://blast.ncbi.nlm.nih.gov/Blast.cgi#alnHdr_1002614778) | *F. solani* | plant pathogen |
| R26 | Ascomycota | [Sordariomycetes](https://de.wikipedia.org/wiki/Sordariomycetes) | [Hypocreales](http://www.mycobank.org/BioloMICS.aspx?TableKey=14682616000000067&Rec=92466&Fields=All) | incertae sedis | *Ilyonectria* | *I. macrodidyma* | plant pathogen |
| R87 | Ascomycota | [Sordariomycetes](https://ceb.wikipedia.org/wiki/Sordariomycetes) | [Hypocreales](http://www.mycobank.org/BioloMICS.aspx?TableKey=14682616000000067&Rec=92466&Fields=All) | incertae sedis | *Sarocladium* | *S. kiliense* | plant associated (endophytic or pathogen) |
| R3 | Ascomycota | Dothideomycetes | Pleosporales | Pleosporaceae | *Alternaria* | *A. alternata* | potential plant pathogen |
| R19 | Ascomycota | Dothideomycetes | Pleosporales | Leptosphaeriaceae | *Leptosphaeria* | *L. biglobosa* | plant pathogen |
| R21 | Ascomycota | Dothideomycetes | Pleosporales | Cucurbitariaceae | *Pyrenochaeta* | *Pyrenochaeta sp* | plant pathogen |
| R18 | Ascomycota | Dothideomycetes | Pleosporales | Phaeosphaeriaceae | *Paraphoma* | *Paraphoma sp* | plant endophyte |
| R81 | Ascomycota | [Sordariomycetes](https://en.wikipedia.org/wiki/Sordariomycetes) | [Glomerellales](http://www.mycobank.org/BioloMICS.aspx?TableKey=14682616000000067&Rec=463582&Fields=All) | Plectosphaerellaceae | *Plectosphaerella* | *P. cucumerina* | plant pathogen |
| R77 | Basidiomycota | Agaricomycetes | Polyporales | Polyporaceae | *Trametes* | *T. versicolor* | saprophyte |
| r15 | Ascomycota | [Sordariomycetes](https://sv.wikipedia.org/wiki/Sordariomycetes) | [Sordariales](https://sv.wikipedia.org/wiki/Sordariales) | [Chaetomiaceae](https://sv.wikipedia.org/wiki/Chaetomiaceae) | *Chaetomium* | *C. funicola/arxii/indicum* | plant endophyte |
